# A checklist of the Ukrainian Xoridinae (Hymenoptera, Ichneumonidae)

**DOI:** 10.3897/BDJ.3.e4832

**Published:** 2015-09-24

**Authors:** Oleksandr Varga

**Affiliations:** ‡ I.I. Schmalhausen Institute of Zoology, NAS of Ukraine, Kiyv, Ukraine

**Keywords:** Parasitoids, Ichneumonidae, Xoridinae, Ukraine, checklist, new records

## Abstract

The Ukrainian Xoridinae list containing 28 species is reviewed. Four species, *Xorides flavotibialis* Hilszczanski, 2000, *X. hedwigi* Clement, 1938, *Xorides rufipes* (Gravenhorst, 1829), and *X. rusticus* (Desvignes, 1856) are recorded in the Ukrainian fauna for the first time. *Agrilus biguttatus* F. is recorded as a host of *Ischnoceros caligatus* (Gravenhorst, 1829) for the first time.

## Introduction

The Xoridinae Shuckard,1840 is a relatively small subfamily of Ichneumonidae, with 220 described species worldwide and 46 species in Europe (Yu et al. 2012), classified into four genera (Townes 1969, Wahl 1997). Three of the genera, *Ischnoceros* Gravenhorst, 1829, *Odontocolon* Cushmen, 1942 and *Xorides* Latreille, 1809, occur in Ukraine (Kasparyan 1981, Meyer 1934, Varga 2014a, Varga 2014b).

Xoridines are most commonly encountered in mature forests where they search for larvae of wood-boring Coleoptera. Host records include Cerambycidae and Buprestidae species, associated with coniferous and deciduous forests (Kasparyan 1981, Yu et al. 2012).

The first Ukrainian Xoridinae list, provided by Meyer (1934), included 10 species, mainly recorded from Central and South-Eastern Ukraine. About a half a century later, Kasparyan (1981) prodived new data about the distribution of xoridines in Ukraine. In this work, what is today Ukraine was marked as "South" and "South-West" (and "West" together with Belarus) of the European part of the former USSR. In most cases the records are from Eastern Ukraine, but they lack specific locality information (only in some cases the author provided a clear locality name, e.g. Kharkiv). Western Ukraine, especially the Carpathians, remained largely unstudied for xoridines until 2014. Recent research carried out by the author demonstrated high Xoridinae species richness in the region (Varga 2014a, Varga 2014b).

The main goal of this paper is to provide summarized data on the distribution of the subfamily Xoridinae in Ukraine, which will provide a basis for a future revision of this group.

## Materials and methods

**Figure 1.**
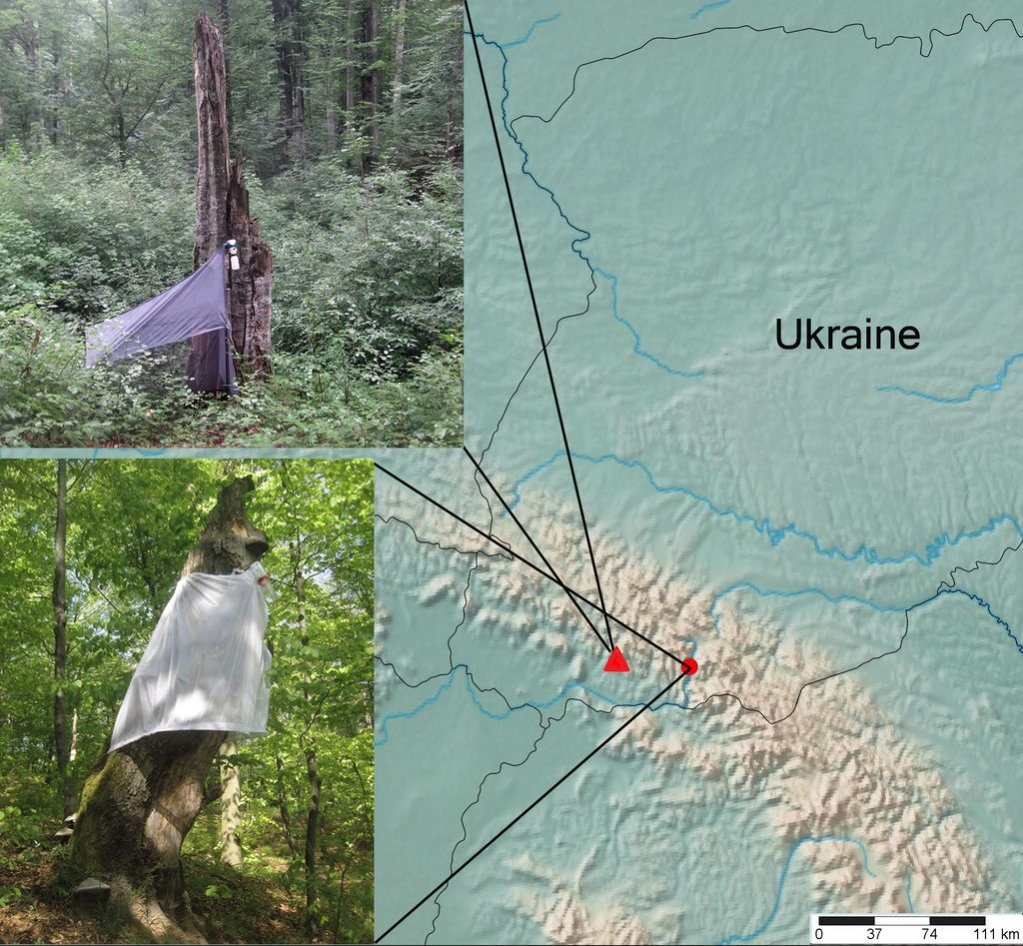
Some of the trapping sites in the Carpathians: triangle - Malaise trap in the primeval beech forest near the village of Mala Ugolka; circle - Tereshkin trap on a dead *Fagus sylvatica* trunk near the village of Kvasy.

This study is based on specimens collected by sweep netting and Malaise (Fig. 1) and conical traps (Fig. 1) (so-called Tereshkin′s traps, see Tereshkin (1990)). The sampling was done by the author in the Ukrainian Carpathians in 2014 and 2015. The specimens are deposited in the Schmalhausen Institute of Zoology (Kiyv, Ukraine). Material from the Zoological Institute of St Petersburg, Russia, was also studied. The images were taken at the Alexandru Ioan Cuza University (Iasi, Romania) using a Leica stereomicroscope 205A with DFC 500 camera, combined with Zerene® software. The species were identified using keys provided by Kasparyan (1981) and Hilszczanski (2000). The images of most of the species were sent to Jacek Hilszczanski to confirm their identification.​ General distribution of species follows Yu et al. (2012).

## Taxon treatments

### Ischnoceros caligatus

(Gravenhorst, 1829)

#### Materials

- **Type status:** Other material. **Occurrence:** recordedBy: V. Kozak; individualCount: 1; sex: female; lifeStage: adult; **Location:** country: Ukraine; stateProvince: Volyn Region; **Identification:** identifiedBy: O. Varga; dateIdentified: 2013; **Event:** samplingProtocol: reared ex. *Agrilus biguttatus* F.; eventDate: 08/07/1976
- **Type status:** Other material. **Occurrence:** recordedBy: A. Gezun; individualCount: 1; sex: female; lifeStage: adult; **Location:** country: Ukraine; stateProvince: Kiyv Region; locality: Batieva Gora; **Identification:** identifiedBy: O. Varga; dateIdentified: 2013; **Event:** samplingProtocol: sweeping; eventDate: 05/28/2007

#### Distribution

**Figure 2.**
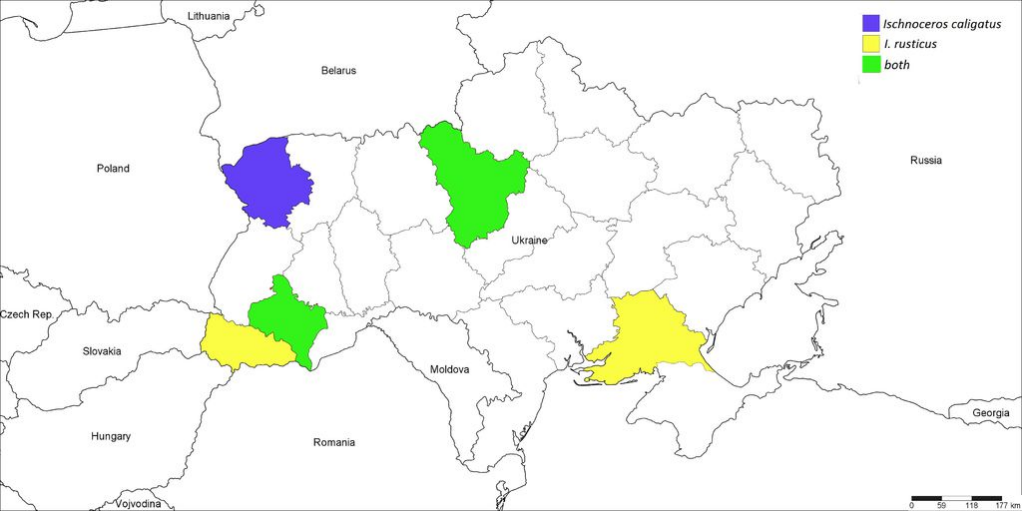
The Ukrainian distribution map of *Ischnoceros caligatus* (Gravenhorst, 1829) and *I. rusticus* (Geoffroy, 1785).

Palaearctic (Yu et al. 2012); Ukraine (Fig. 2): Ivano-Frankivsk Region (Varga 2014a), Volyn and Kyiv Regions.<br/>

#### Notes

*Agrilus biguttatus* F. (Coleoptera: Buprestidae) is recorded as a host of this species for the first time.

### Ischnoceros rusticus

(Geoffroy, 1785)

#### Materials

- **Type status:** Other material. **Occurrence:** recordedBy: M. Nesterov; individualCount: 1; sex: female; lifeStage: adult; **Location:** country: Ukraine; stateProvince: Kiyv Region; locality: Hodosovka; **Identification:** identifiedBy: O. Varga; dateIdentified: 2013; **Event:** samplingProtocol: sweeping; eventDate: 04/24/2004
- **Type status:** Other material. **Occurrence:** recordedBy: O. Varga; individualCount: 1; sex: female; lifeStage: adult; **Location:** country: Ukraine; stateProvince: Transcarpathian Region; county: Tyachiv District; locality: Carpathian Biosphere Reserve, prymeval beech forest near Mala Ugolka; verbatimElevation: 750 m; verbatimCoordinates: 48°15'39.58"N, 23°37'0.84"E; **Identification:** identifiedBy: O. Varga; dateIdentified: 2014; **Event:** samplingProtocol: Malaise trap; startDayOfYear: 07/17/2014; endDayOfYear: 08/16/2014

#### Distribution

Palaearctic (Yu et al. 2012); Ukraine (Fig. 2): ​Kherson Region (Kasparyan 1981​), Ivano-Frankivsk Region (Varga 2014a), Kyiv and Transcarpathian Regions.

### Odontocolon dentipes

(Gmelin, 1790)

#### Materials

- **Type status:** Other material. **Occurrence:** recordedBy: M. Nesterov; individualCount: 1; sex: male; lifeStage: adult; **Location:** country: Ukraine; stateProvince: Kiyv Region; locality: Belitchi; **Identification:** identifiedBy: O. Varga; dateIdentified: 2013; **Event:** samplingProtocol: sweeping; eventDate: 06/01/2006
- **Type status:** Other material. **Occurrence:** recordedBy: Paramonov; individualCount: 1; sex: female; lifeStage: adult; **Location:** country: Ukraine; stateProvince: Kiyv Region; locality: Kiyv; **Identification:** identifiedBy: O. Varga; dateIdentified: 2013; **Event:** samplingProtocol: sweeping; eventDate: 03/21/1924
- **Type status:** Other material. **Occurrence:** recordedBy: O. Varga; individualCount: 1; sex: male; lifeStage: adult; **Location:** country: Ukraine; stateProvince: Transcarpathian Region; county: Rakhiv District; locality: Carpathian Biosphere Reserve, Chornogora, slopes of m. Sheshul; verbatimElevation: 1450 m; verbatimCoordinates: 48°09'25.05"N, 24°20'59.53"E; **Identification:** identifiedBy: O. Varga; dateIdentified: 2014; **Event:** samplingProtocol: Malaise trap; eventDate: 2014-06-05/29

#### Distribution

**Figure 3.**
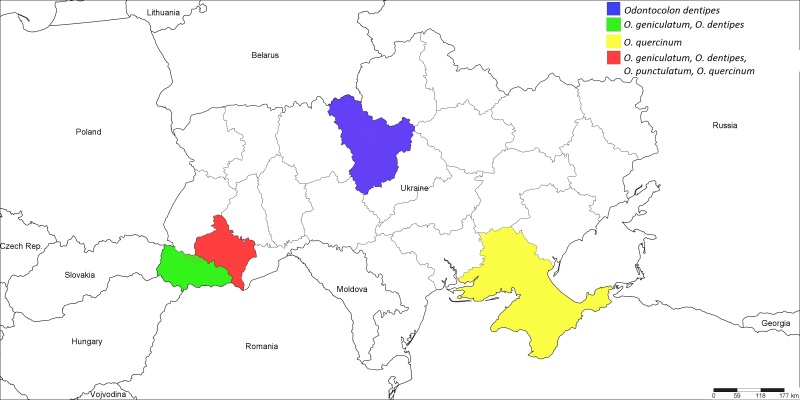
The Ukrainian distribution map of *Odontocolon dentipes* (Gmelin, 1790), *O. geniculatum* (Kriechbaumer, 1889), *O. punctulatum* (Thomson, 1877), and *O. quercinum* (Thomson, 1877).

Palaearctic (Yu et al. 2012); Ukraine (Fig. 3): ​Ivano-Frankivsk Region (Varga 2014a), Kiyv and Transcarpathian Regions.

### Odontocolon geniculatum

(Kriechbaumer, 1889)

#### Distribution

Palaearctic (Yu et al. 2012); Ukraine (Fig. 3): ​Ivano-Frankivsk and Transcarpathian Regions (Varga 2014a).

### Odontocolon punctulatum

(Thomson, 1877)

#### Distribution

Western Palaearctic (Yu et al. 2012); Ukraine (Fig. 3): ​Ivano-Frankivsk Region (Varga 2014a).

### Odontocolon quercinum

(Thomson, 1877)

#### Materials

- **Type status:** Other material. **Occurrence:** recordedBy: A. Kotenko; individualCount: 1; sex: male; lifeStage: adult; **Location:** country: Ukraine; stateProvince: Crimea; county: Balaclava District; locality: Aya Cape; **Identification:** identifiedBy: O. Varga; dateIdentified: 2013; **Event:** samplingProtocol: sweeping; eventDate: 07/22/1979

#### Distribution

Western Palaearctic (Yu et al. 2012); Ukraine (Fig. 3): Kherson Region (Kasparyan 1981), Ivano-Frankivsk Region (Varga 2014a), Crimea.

### Odontocolon rufiventris

(Holmgren, 1860)

#### Materials

- **Type status:** Other material. **Occurrence:** recordedBy: O. Varga; individualCount: 1; sex: female; lifeStage: adult; **Location:** country: Ukraine; stateProvince: Transcarpathian Region; county: Tyachiv District; locality: Carpathian Nature Reserve, beech forest, 6.5 km N of Mala Ugolka; verbatimElevation: 750 m; verbatimCoordinates: 48°15'39.58"N, 23°37'0.84"E; **Identification:** identifiedBy: O. Varga; dateIdentified: 2015; **Event:** samplingProtocol: Malaise trap; eventDate: 2015-05-12/31

#### Distribution

**Figure 4.**
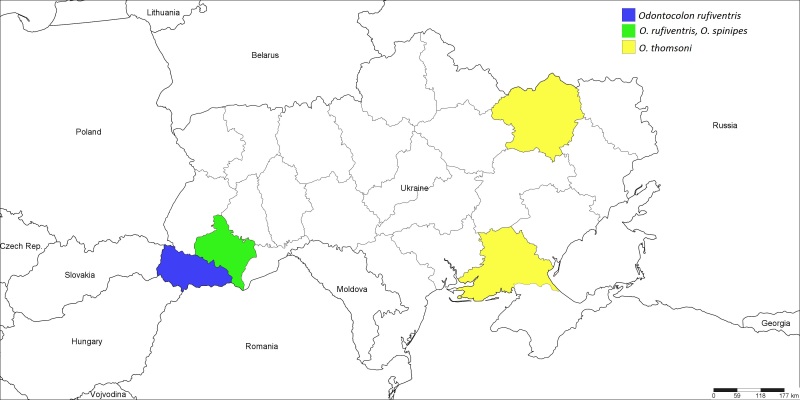
The Ukrainian distribution map of *Odontocolon rufiventris* (Holmgren, 1860), *O. spinipes* (Gravenhorst, 1829), and *O. thomsoni* (Clement, 1938).

Western Palaearctic (Yu et al. 2012); Ukraine (Fig. 4): ​Ivano-Frankivsk Region (Varga 2014a), Transcarpathian Region.

### Odontocolon spinipes

(Gravenhorst, 1829)

#### Distribution

Palaearctic (Yu et al. 2012); Ukraine (Fig. 4): ​Ivano-Frankivsk Region (Varga 2014a).

### Odontocolon thomsoni

(Clément, 1938)

#### Distribution

Western Palaearctic (Yu et al. 2012); Ukraine (Fig. 4): ​Kharkiv and Kherson Regions (Kasparyan 1981).

### Xorides alpestris

(Habermehl, 1903)

#### Materials

- **Type status:** Other material. **Occurrence:** recordedBy: M. Nesterov; individualCount: 1; sex: male; lifeStage: adult; **Location:** country: Ukraine; stateProvince: Kiyv Region; locality: Belitchi; **Identification:** identifiedBy: O. Varga; dateIdentified: 2013; **Event:** samplingProtocol: sweeping; eventDate: 06/01/2006

#### Distribution

**Figure 5.**
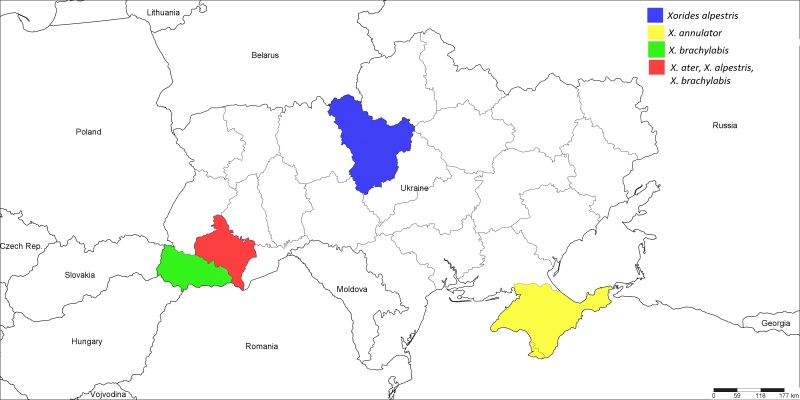
The Ukrainian distribution map of *Xorides alpestris* (Habermehl, 1903), *X. annulator* (Fabricius, 1804), *X. ater* (Gravenhorst, 1829), and *X. brachylabis* (Kriechbaumer, 1889).

Palaearctic (Yu et al. 2012); Ukraine (Fig. 5): ​Ivano-Frankivsk Region (Varga 2014b) and Kiyv Region.

### Xorides annulator

(Fabricius, 1804)

#### Distribution

Palaearctic (Yu et al. 2012); Ukraine (Fig. 5): ​Crimea (Kasparyan 1981).

#### Notes

There is only one specimen with a very old and illegible label, and itseems to be collected (as the other species in the collection) from South-East Ukraine. Therefore, the data provided is from the literature.

### Xorides ater

(Gravenhorst, 1829)

#### Distribution

Palaearctic (Yu et al. 2012); Ukraine (Fig. 5): South-East Ukraine (Kasparyan 1981), Ivano-Frankivsk Region (Varga 2014b).

#### Notes

There is only specimen with a very old and illegible label, and itseems to be collected (as the other species in the collection) from South-East Ukraine. Therefore, the data provided is from the literature.

### Xorides brachylabis

(Kriechbaumer, 1889)

#### Distribution

Palaearctic (Yu et al. 2012); Ukraine (Fig. 5): ​Ivano-Frankivsk and Transcarpathian Regions (Varga 2014b).

### Xorides csikii

Clement, 1938

#### Materials

- **Type status:** Other material. **Occurrence:** recordedBy: M. Nesterov; individualCount: 1; sex: female; lifeStage: adult; **Location:** country: Ukraine; stateProvince: Kiyv Region; locality: Hodosovka; **Identification:** identifiedBy: O. Varga; dateIdentified: 2013; **Event:** samplingProtocol: sweeping; eventDate: 05/06/2005

#### Distribution

**Figure 6.**
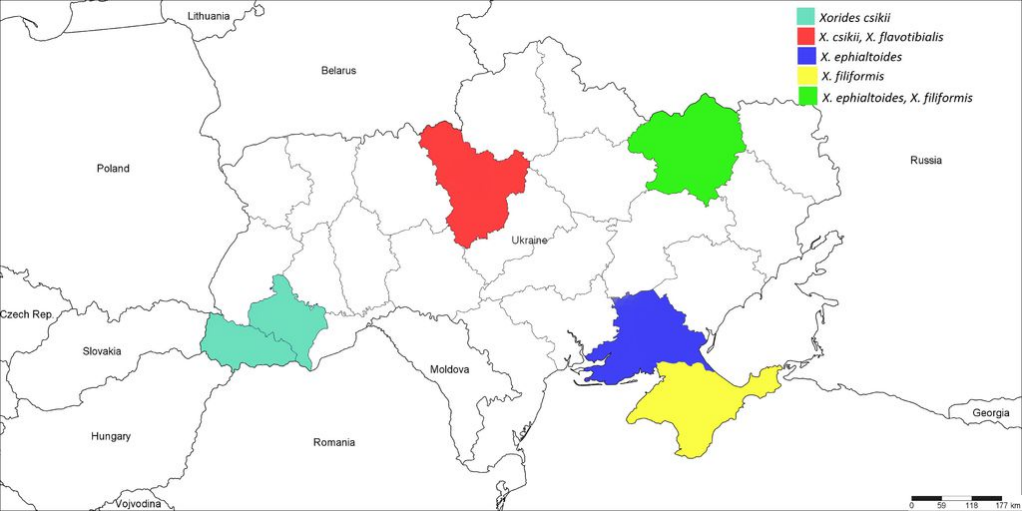
The Ukrainian distribution map of *Xorides csikii* Clement, 1938, *X. ephialtoides* (Kriechbaumer, 1882), *X. filiformis* (Gravenhorst, 1829), and *X. flavotibialis* Hilszczanski, 2000.

Western Palaearctic, previously recorded only from France, Germany, Hungary, Poland, Switzerland, and United Kingdom (Yu et al. 2012); Ukraine (Fig. 6): ​Ivano-Frankivsk and Transcarpathian Regions (Varga 2014b), Kiyv Region.

### Xorides ephialtoides

(Kriechbaumer, 1882)

#### Distribution

Palaearctic (Yu et al. 2012); Ukraine (Fig. 6): ​Kharkiv and Kherson Regions (Kasparyan 1981).

### Xorides filiformis

(Gravenhorst, 1829)

#### Materials

- **Type status:** Other material. **Occurrence:** recordedBy: A. Sirenko; individualCount: 1; sex: female; lifeStage: adult; **Location:** country: Ukraine; stateProvince: Crimea; locality: Karabi Yayla; **Identification:** identifiedBy: O. Varga; dateIdentified: 2012; **Event:** samplingProtocol: sweeping; eventDate: 2003

#### Distribution

Western Palaearctic (Yu et al. 2012); Ukraine (Fig. 6): Kharkiv Region (Kasparyan 1981), Crimea.

### Xorides flavotibialis

Hilszczanski, 2000

#### Materials

- **Type status:** Other material. **Occurrence:** recordedBy: A. Gesun; individualCount: 1; sex: female; lifeStage: adult; **Location:** country: Ukraine; stateProvince: Kyiv Region; locality: Batyeva Gora; **Identification:** identifiedBy: O. Varga; dateIdentified: 2014; **Event:** samplingProtocol: sweeping; eventDate: 06/10/2007

#### Distribution

**Figure 7.**
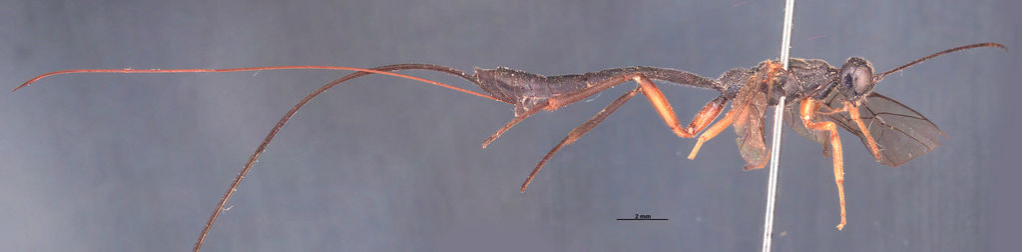
*Xorides flavotibialis* Hilszczanski, 2000.

This species is known only from Poland (Hilszczanski 2000); Ukraine (Fig. 6): ​Kiyv Region, new for Ukraine (Fig. 7).

### Xorides gravenhorstii

(Curtis, 1831)

#### Materials

- **Type status:** Other material. **Occurrence:** individualCount: 1; sex: male; lifeStage: adult; **Location:** country: Ukraine; stateProvince: Crimea; locality: Kanaka; **Identification:** identifiedBy: O. Varga; dateIdentified: 2013; **Event:** samplingProtocol: sweeping; eventDate: 05/13/1993

#### Distribution

**Figure 8.**
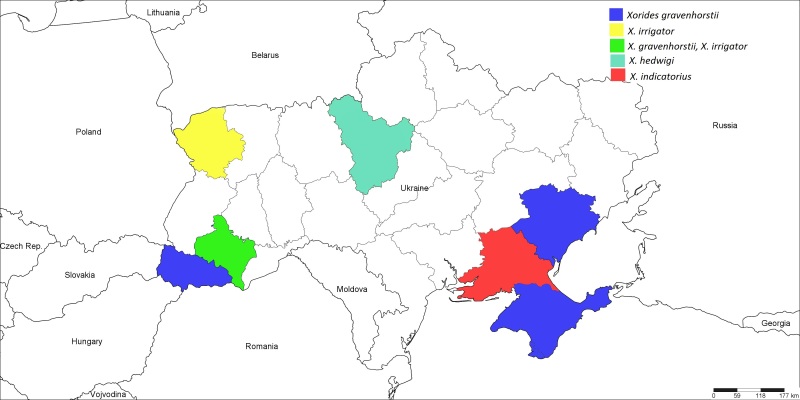
The Ukrainian distribution map of *Xorides gravenhorstii* (Curtis, 1831), *X. hedwigi* Clement, 1938, *X. indicatorius* (Latreille, 1806), and *X. irrigator* (Fabricius, 1793).

Western Palaearctic (Yu et al. 2012); Ukraine (Fig. 8): Crimea, Zaporyzhzhya Region (Kasparyan 1981), Ivano-Frankivsk and Transcarpathian Regions (Varga 2014b).

### Xorides hedwigi

(Clement, 1938)

#### Materials

- **Type status:** Other material. **Occurrence:** recordedBy: A. Kotenko; individualCount: 1; sex: female; lifeStage: adult; **Location:** country: Ukraine; stateProvince: Kyiv Region; locality: Novosilky; **Identification:** identifiedBy: O. Varga; dateIdentified: 2014; **Event:** samplingProtocol: sweeping; eventDate: 06/07/1993

#### Distribution

**Figure 9.**
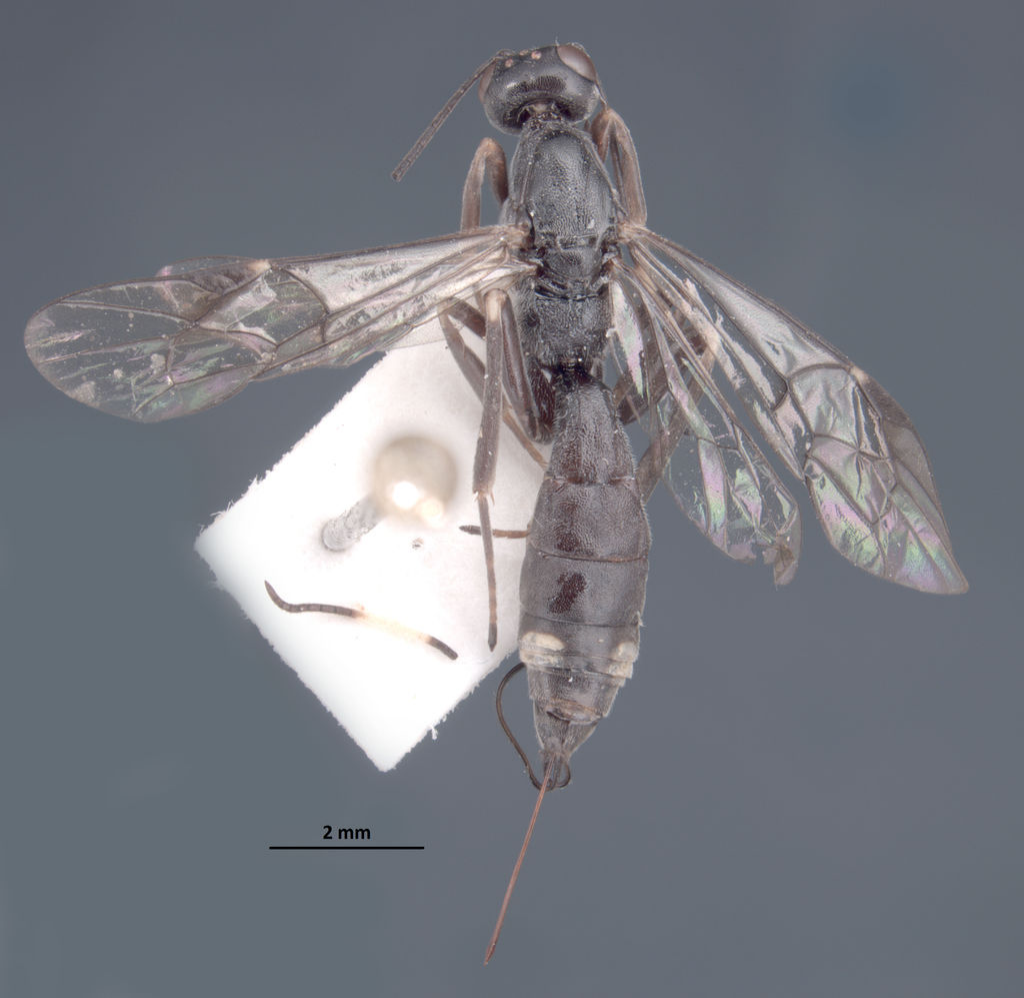
*Xorides hedwigi* Clement, 1938.

Palaearctic, recorded only from Austria, China, Czech Republic, former Czechoslovakia, Germany, Hungary, and Poland (Yu et al. 2012); Ukraine (Fig. 8): ​Kyiv Region, new for Ukraine (Fig. 9).

### Xorides indicatorius

(Latreille, 1806)

#### Distribution

Western Palaearctic (Yu et al. 2012); Ukraine (Fig. 8): Kherson Region (Kasparyan 1981).

### Xorides irrigator

(Fabricius, 1793)

#### Materials

- **Type status:** Other material. **Occurrence:** individualCount: 2; sex: females; lifeStage: adult; **Location:** country: Ukraine; stateProvince: Volyn Region; county: Lyubomlsk District; **Identification:** identifiedBy: D. Kasparyan; dateIdentified: 1977; **Event:** samplingProtocol: reared ex. *Acanthocinus aedilis* L.; eventDate: 08/01/1975

#### Distribution

Palaearctic (Yu et al. 2012); Ukraine (Fig. 8): ​Ivano-Frankivsk Region (Varga 2014b), Volyn Region.

### Xorides niger

(Pfeffer, 1913)

#### Distribution

**Figure 10.**
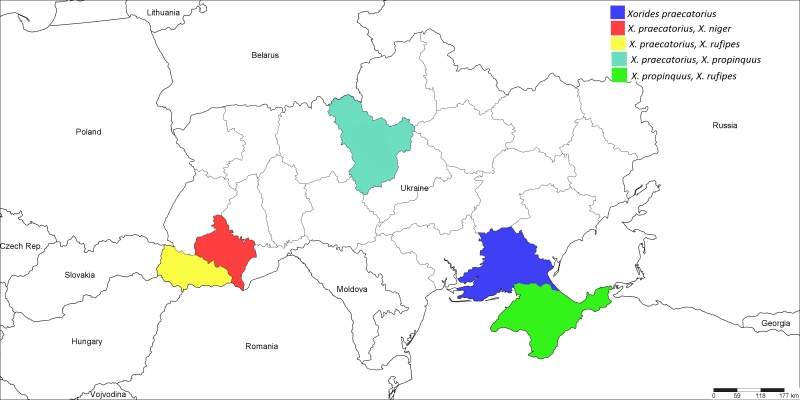
The Ukrainian distribution map of *Xorides niger* (Pfeffer, 1913), *X. praecatorius* (Fabricius, 1793), *X. propinquus* (Tschek, 1869), and *X. rufipes* (Gravenhorst, 1829).

Western Palaearctic (Yu et al. 2012); Ukraine (Fig. 10): ​Ivano-Frankivsk Region (Varga 2014b).

### Xorides praecatorius

(Fabricius, 1793)

#### Materials

- **Type status:** Other material. **Occurrence:** recordNumber: A. Prohorov; individualCount: 1; sex: male; lifeStage: adult; **Location:** country: Ukraine; stateProvince: Kiyv Region; locality: Novobelitchi; **Identification:** identifiedBy: Oleksandr Varga; dateIdentified: 2013; **Event:** samplingEffort: sweeping; eventDate: 04/30/2010
- **Type status:** Other material. **Occurrence:** recordNumber: M. Nesterov; individualCount: 1; sex: male; lifeStage: adult; **Location:** country: Ukraine; stateProvince: Kiyv Region; locality: Hodosovka; **Identification:** identifiedBy: Oleksandr Varga; dateIdentified: 2013; **Event:** samplingEffort: sweeping; eventDate: 05/06/2005
- **Type status:** Other material. **Occurrence:** recordNumber: A. Gezun; individualCount: 1; sex: male; lifeStage: adult; **Location:** country: Ukraine; stateProvince: Kiyv Region; locality: Batieva Gora; **Identification:** identifiedBy: Oleksandr Varga; dateIdentified: 2013; **Event:** samplingEffort: sweeping; eventDate: 05/29/2007

#### Distribution

Palaearctic (Yu et al. 2012); Ukraine (Fig. 10): Kherson Region (Kasparyan 1981), Ivano-Frankivsk and Transcarpathian Regions (Varga 2014b), Kiyv Region.

### Xorides propinquus

(Tschek, 1869)

#### Materials

- **Type status:** Other material. **Occurrence:** recordedBy: A. Gesun; individualCount: 1; sex: female; lifeStage: adult; **Location:** country: Ukraine; stateProvince: Kyiv Region; locality: Batieva Gora; **Identification:** identifiedBy: O. Varga; dateIdentified: 2014; **Event:** samplingProtocol: sweeping; eventDate: 05/27/2007

#### Distribution

Palaearctic (Yu et al. 2012); Ukraine (Fig. 10): Crimea (Kasparyan 1981), Kyiv Region.

### Xorides rufipes

(Gravenhorst, 1829)

#### Materials

- **Type status:** Other material. **Occurrence:** recordedBy: O. Varga; individualCount: 2; sex: females; lifeStage: adult; **Location:** country: Ukraine; stateProvince: Transcarpathian Region; county: Rakhiv District; locality: Carpathian Biosphere Reserve, Svydovets, beech forest 2-3 km NW of Kvasy; verbatimElevation: 850 m; verbatimCoordinates: 48°09'08.89"N, 24°15'58.35"E; **Identification:** identifiedBy: O. Varga; dateIdentified: 2014; **Event:** samplingProtocol: Trunk trap; startDayOfYear: 05/07/2014; endDayOfYear: 06/05/2014
- **Type status:** Other material. **Occurrence:** recordedBy: A. Kotenko; individualCount: 1; sex: female; lifeStage: adult; **Location:** country: Ukraine; stateProvince: Crimea; county: Balaklava District; locality: Aya Cape; **Identification:** identifiedBy: O. Varga; dateIdentified: 2014; **Event:** samplingProtocol: sweeping; eventDate: 07/22/1979
- **Type status:** Other material. **Occurrence:** recordedBy: O. Varga; individualCount: 1; sex: female; lifeStage: adult; **Location:** country: Ukraine; stateProvince: Transcarpathian Region; county: Tyachiv District; locality: Carpathian Nature Reserve, beech forest, 6.5 km N of Mala Ugolka; verbatimElevation: 750 m; verbatimCoordinates: 48°15'39.58"N, 23°37'0.84"E; **Identification:** identifiedBy: O. Varga; dateIdentified: 2015; **Event:** samplingProtocol: near dead *Fagus sylvatica* trunk; eventDate: 05/31/2015
- **Type status:** Other material. **Occurrence:** recordedBy: O. Varga; individualCount: 2; sex: females; lifeStage: adult; **Location:** country: Ukraine; stateProvince: Transcarpathian Region; county: Vynogradiv District; locality: Carpathian Nature Reserve, Vynogradiv, oak forest, Chorna Gora; verbatimElevation: 500 m; verbatimCoordinates: 48°09'19.70"N, 23°04'22.47"E; **Identification:** identifiedBy: O. Varga; dateIdentified: 2015; **Event:** samplingProtocol: near dead oak trunk; eventDate: 05/30/2015
- **Type status:** Other material. **Occurrence:** recordedBy: O. Varga; individualCount: 1; sex: male; lifeStage: adult; **Location:** country: Ukraine; stateProvince: Transcarpathian Region; county: Vynogradiv District; locality: Carpathian Nature Reserve, Vynogradiv, oak forest, Chorna Gora; verbatimElevation: 500 m; verbatimCoordinates: 48°09'19.70"N, 23°04'22.47"E; **Identification:** identifiedBy: O. Varga; dateIdentified: 2015; **Event:** samplingProtocol: near dead oak trunk; eventDate: 05/30/2015

#### Distribution

Palaearctic (Yu et al. 2012); Ukraine (Fig. 10): Transcarpathian Region and Crimea, new for Ukraine.

### Xorides rusticus

(Desvignes, 1856)

#### Materials

- **Type status:** Other material. **Occurrence:** recordedBy: A. Kotenko; individualCount: 1; sex: female; lifeStage: adult; **Location:** country: Ukraine; stateProvince: Kyiv Region; locality: Kyiv, Feofania; **Identification:** identifiedBy: O. Varga; dateIdentified: 2014; **Event:** samplingProtocol: sweeping; eventDate: 06/19/2002

#### Distribution

**Figure 11.**
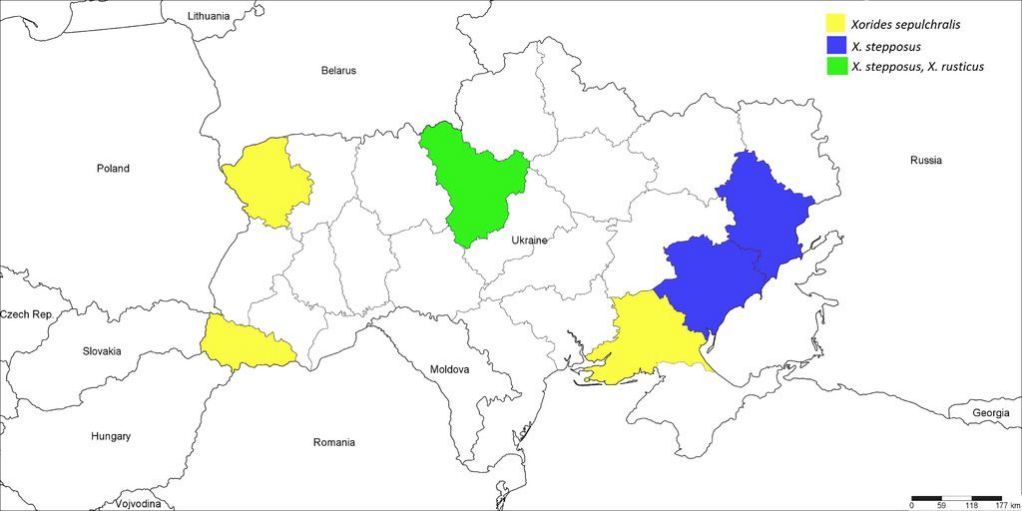
The Ukrainian distribution map of *Xorides rusticus* (Desvignes, 1856), *X. sepulchralis* (Holmgren, 1860), and *X. stepposus* Kasparyan, 1981.

Palaearctic, previously recorded only from China, Germany, Poland, and United Kingdom (Yu et al. 2012); Ukraine (Fig. 11): ​Kyiv Region, new for Ukraine.

### Xorides sepulchralis

(Holmgren, 1860)

#### Materials

- **Type status:** Other material. **Occurrence:** recordedBy: A. Ermolenko; individualCount: 1; sex: female; lifeStage: adult; **Location:** country: Ukraine; stateProvince: Volyn Region; locality: Nevyr; **Identification:** identifiedBy: O. Varga; dateIdentified: 2014; **Event:** samplingProtocol: sweeping; eventDate: 08/03/1960
- **Type status:** Other material. **Occurrence:** recordedBy: O. Varga; individualCount: 3; sex: males; lifeStage: adult; **Location:** country: Ukraine; stateProvince: Transcarpathian Region; county: Vynogradiv District; locality: Carpathian Nature Reserve, Vynogradiv, oak forest, Chorna Gora; verbatimElevation: 500 m; verbatimCoordinates: 48°09'19.70"N, 23°04'22.47"E; **Identification:** identifiedBy: O. Varga; dateIdentified: 2015; **Event:** samplingProtocol: near dead oak trunk; eventDate: 05/30/2015
- **Type status:** Other material. **Occurrence:** recordedBy: O. Varga; individualCount: 1; sex: female; lifeStage: adult; **Location:** country: Ukraine; stateProvince: Transcarpathian Region; county: Tyachiv District; locality: Carpathian Nature Reserve, beech forest, 6.5 km N of Mala Ugolka; verbatimElevation: 750 m; verbatimCoordinates: 48°15'39.58"N, 23°37'0.84"E; **Identification:** identifiedBy: O. Varga; dateIdentified: 2015; **Event:** samplingProtocol: Malaise trap; eventDate: 2015-05-12/31

#### Distribution

**Figure 12.**
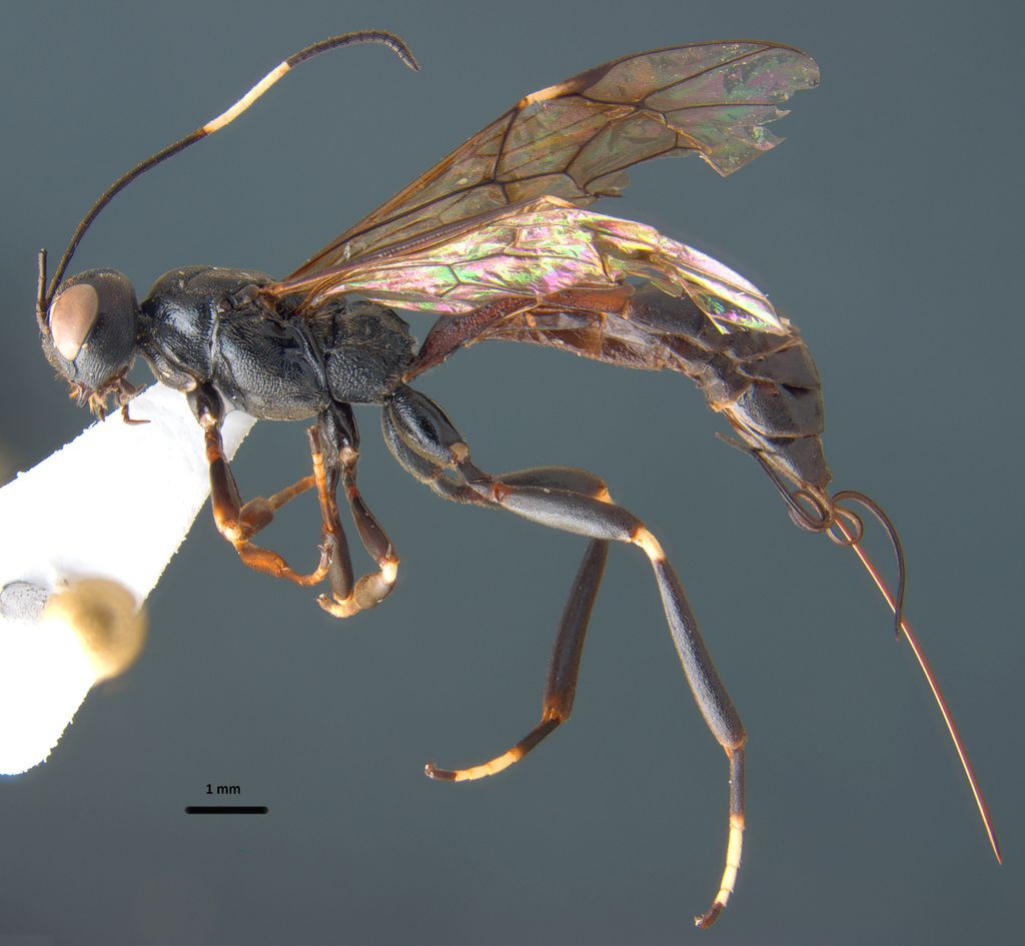
*Xorides sepulchralis* (Holmgren, 1860).

Palaearctic (Yu et al. 2012); Ukraine (Fig. 11): Kherson Region (Kasparyan 1981), Volyn and Transcarpathian Regions (Fig. 12).

### Xorides stepposus

Kasparyan, 1981

#### Materials

- **Type status:** Other material. **Occurrence:** recordedBy: A. Prohorov; individualCount: 1; sex: female; lifeStage: adult; **Location:** country: Ukraine; stateProvince: Kyiv Region; locality: Kyiv, Svyatoshyno; **Identification:** identifiedBy: O. Varga; dateIdentified: 2014; **Event:** samplingProtocol: sweeping; eventDate: 05/04/2011

#### Distribution

**Figure 13.**
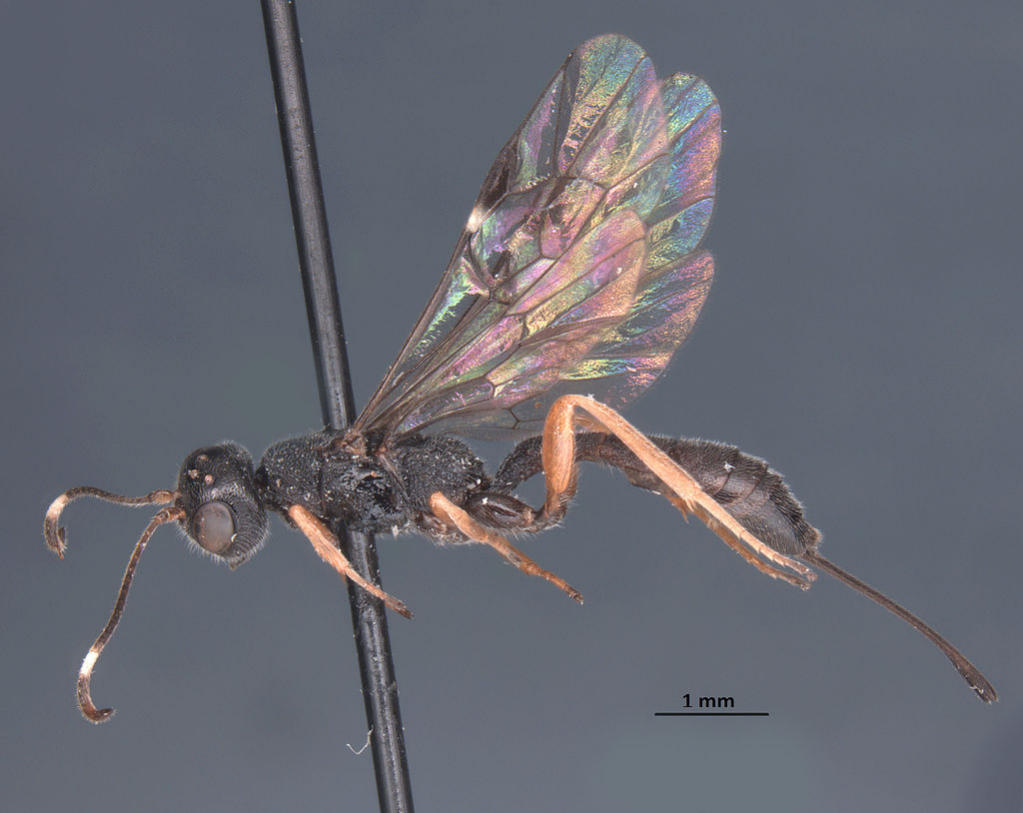
*Xorides stepposus* Kasparyan, 1981.

This species is only known from Ukraine (Fig. 11): ​Donetsk and Zaporizhzhya Regions (Kasparyan 1981), Kiyv Region (Fig. 13).

## Discussion

Before this work, the Ukrainian Xoridinae was a poorly studied group of ichneumonids comprising just 15 species, mainly recorded by Meyer (1934) and Kasparyan (1981). The main collections were deposited in Russian museums. Unfortunately, Meyer’s collection was lost during the Second World War and now none of these records can be considered valid as there are no voucher specimens available. Thus, *Xorides gracilicornis* (Gravenhorst, 1829) recorded by Meyer (1934) from the Kiyv Region should be excluded from the Ukrainian list. The same is true for another species, *X. fuligator* (Thunberg, 1822), which is known only from a questionable record by Besser (1835) from the Volyn Region. The Ukrainian distribution of the other species recorded by Meyer (1934) were confirmed in a later work (Kasparyan 1981). This latter collection is deposited in the Zoological Institute of St Petersburg, Russia, and comprises specimens collected mainly from the South-Eastern part of Ukraine (Crimea, Kherson and Kharkiv Regions). Up to 2014, there was no separate Xoridinae collection in the Schmalhausen Institute of Zoology in Kiyv. Only a few pinned specimens (identified by Dmitriy R. Kasparyan) were located in a box with “mixed” ichneumonids. After the recent investigation of the Carpathian Xoridinae fauna (Varga 2014a, Varga 2014b), this collection was largely replenished, increasing the number of the Ukrainian xoridines to 24 species. In addition, some unidentified and unpinned specimens (collected by other scientists) deposited in the institute's collection were also studied by the author. As a result, four new species, *X. flavotibialis* Hilszczanski, 2000, *X. hedwigi* Clement, 1938, *Xorides rufipes* (Gravenhorst, 1829), and *X. rusticus* (Desvignes, 1856) were added to the Ukrainian list.

To conclude, the Ukrainian fauna of the subfamily Xoridinae now contains 28 species, most of which are widespread and common in Europe, but some of them, e.g. *X. csikii* Clement, 1938, *X. flavotibialis* Hilszczanski, 2000, *X. hedwigi* Clement, 1938, and *X. rusticus* (Desvignes, 1856), and *X. stepposus* Kasparyan, 1981, are quite rare.

## Supplementary Material

XML Treatment for Ischnoceros caligatus

XML Treatment for Ischnoceros rusticus

XML Treatment for Odontocolon dentipes

XML Treatment for Odontocolon geniculatum

XML Treatment for Odontocolon punctulatum

XML Treatment for Odontocolon quercinum

XML Treatment for Odontocolon rufiventris

XML Treatment for Odontocolon spinipes

XML Treatment for Odontocolon thomsoni

XML Treatment for Xorides alpestris

XML Treatment for Xorides annulator

XML Treatment for Xorides ater

XML Treatment for Xorides brachylabis

XML Treatment for Xorides csikii

XML Treatment for Xorides ephialtoides

XML Treatment for Xorides filiformis

XML Treatment for Xorides flavotibialis

XML Treatment for Xorides gravenhorstii

XML Treatment for Xorides hedwigi

XML Treatment for Xorides indicatorius

XML Treatment for Xorides irrigator

XML Treatment for Xorides niger

XML Treatment for Xorides praecatorius

XML Treatment for Xorides propinquus

XML Treatment for Xorides rufipes

XML Treatment for Xorides rusticus

XML Treatment for Xorides sepulchralis

XML Treatment for Xorides stepposus
